# Allocation of Flood Drainage Rights Based on the PSR Model and Pythagoras Fuzzy TOPSIS Method

**DOI:** 10.3390/ijerph17165821

**Published:** 2020-08-11

**Authors:** Dandan Zhang, Juqin Shen, Pengfei Liu, Fuhua Sun

**Affiliations:** 1 Business School, Hohai University, Nanjing 211100, China; 2 Yangtze Institute for Conservation and Development, Hohai University, Nanjing 210098, China; jqshen@hhu.edu.cn; 3 College of Agricultural Engineering, Hohai University, Nanjing 210098, China; 4 School of Instrument Science and Engineering, Southeast University, Nanjing 210096, China; 230208375@seu.edu.cn

**Keywords:** flood drainage rights management, urgency of flood drainage rights allocation, PSR model, Pythagoras fuzzy TOPSIS method, Huai River Basin

## Abstract

To minimize losses caused by flooding of areas in a river basin, flood risk management may sacrifice the interests of some areas. Because of regional differences in natural and urban conditions, rankings of the urgencies of flood drainage rights allocations in different regions are of great practical significance to the realization of optimal allocations and reduction of damages. Based on the “pressure–state–response” (PSR) framework, this study designed an index system of flood drainage rights allocations in river basins for the comprehensive consideration of the different attributes of regional societies, environments, and technologies, as well as the differences in the quality of technical management and in the levels of social and economic development. A Pythagoras fuzzy TOPSIS method was used to evaluate the urgencies and determine the management of allocations in different areas. Eight cities in Jiangsu Province in the Huai River Basin were selected as the research objects. The results showed that pressure factors played dominant roles in the degrees of urgency. Among the cities, Nantong had the highest degree, followed by Taizhou, whereas Lianyungang had the lowest. The degrees in the central region of Jiangsu were higher than in the northern region.

## 1. Introduction

The global climate is undergoing significant changes characterized by warming. The increasing trend of extreme droughts and floods caused by climate warming has become the focus of attention of all countries [1,2]. Among them, the impact of flood is extensive. Flood is the result of the interaction between natural factors and human activities [3]. Within a river basin, the upper, middle, and lower reaches, as well as the tributaries and main streams, have the same environmental characteristics and internal relations. Natural disasters, especially floods, would directly affect the development of the whole basin [4,5]. In a flood, the water level of the river system rises and the drainage demand of each region increases. Meanwhile, secondary disasters would rapidly affect social and economic conditions. For example, the Brahmaputra River Basin in northeastern India is famous for its abundant water and hydropower resources, as well as for its frequent floods during every rainy season, which cause massive economic losses and casualties along the banks [6,7]. The resulting losses in food production affect India’s economy significantly [8]. Increasing population and economic development have contributed to difficulties in integrated watershed and flood management, as well as given rise to disputes over water resources allocation [9].

Different subjects in a river basin give rise to different demands that lead to flood drainage conflicts and affect the interests of the populaces in the lower reaches of the river [10]. For example, after the 2011catastrophic floods in Bangkok, Thailand, the local public clashed with the government over the latter’s decision to open upstream sluice gates to prevent the flood from affecting the city center [11,12,13]. When Devil’s Lake in North Dakota, United States, floods, artificial drainage is usually used to discharge water into the adjacent river system. This strategy has drawn people’s attention to the water quality and downstream floods, as well as the cross-border drainage conflicts between the United States and Canada [14,15]. The concept of integrated flood management was proposed as early as 2002. Since then, scholars have proposed multidisciplinary studies on the methods of such management [16]. Within the context of integrated water resources management (IWRM), the benefits of flood zones have been maximized and damages have been reduced by the integrated development of land and water resources within river basins [17].

Floods are responsible for 60% of the global annual natural disaster losses. China suffers the most serious flood disasters in the world [18]. According to incomplete statistics, more than 400 disputes from 1949 to 1985 had been caused by flood drainage in the Huai River Basin [19]. Although China’s flood control engineering measures have been continuously strengthened since 1985, the supplement of non-engineering measures is also necessary, and basin flood management is imminent. Some areas consider only their own interests and damage the interests of the whole basin, so the watershed management department should consider the drainage capacities of water conservancy projects in the basin, as well as consider the rainfall distribution and drainage demand in each region. Combined with the social, economic, and ecological environments of each region, the rights of flood drainage to the water conservancy projects in each region are arranged in order to reduce damage within the basin [20]. When flood dispatching is conducted within the basin, sometimes the interests of some areas may be sacrificed for the sake of flood discharges, and the optimal allocations of drainage rights can be facilitated through appropriate economic compensations. As an important non-engineering measure, the management of flood drainage rights can minimize the loss of inundation in the downstream, alleviate drainage contradiction and water disputes, realize the maximum benefits of flood control in the basin, and ensure social and economic development.

When the safe displacements of water conservancy projects in the whole basin are limited and the total demand for drainage exceeds the displacements, the flood control pressures on the watershed management department are enormous [21,22]. Because of regional differences in urbanization, emergency treatments, and flood control water levels, it is the most intuitive way to rank the urgencies of flood drainage demands in different regions [23]. Low priorities of the flood drainage rights in the study area indicate that it had been greatly affected by floods, so the distribution of flood drainage right should be given priority; otherwise, the area had not been so affected by flood disasters and the drainage demand was low. Therefore, the determination of the priorities of different areas to obtain flood drainage rights has become an urgent problem. This study analyzed the factors that affect the allocations of flood drainage rights, for which it has established an evaluation index system. Because the process of flood drainage rights allocation is a multi-objective complex system, the application of a combination of Pythagorean fuzzy set and TOPSIS methods can be used to measure the urgencies and rank them.

## 2. Literature Review

Assessments of flood disasters, vulnerability, and integrated risks are the main concerns of flood risk management [24,25,26]. By combining stakeholder theory and multi-criteria assessment in a geographic information system (GIS), Hazarika, N. et al. evaluated the vulnerability of the upper reaches of the Brahmaputra River to flooding [27]. Using the fuzzy VIKOR Method, Lee et al. quantified spatial flood vulnerability in a multi-criterion manner [28]. Flood prediction is also one of the focuses of research [29]. Considering that downstream countries were vulnerable to flood disasters as a result of the lack of hydrological and meteorological information in upstream countries, Sikder et al. simulated the hydrological changes of cross-border basins through numerical models and compared the simulation results of a land surface model (SLM) with historical flow to evaluate the quality of runoff data [30]. Wang et al. used a Burg entropy spectral analysis model (BESA) trained by remote sensing data to predict the runoffs during flood seasons and predicted the annual variation trends of rice and tea in affected areas [31]. In addition, some scholars, such as Sarmah and Das, have discussed methods of urban flood control planning and designed a comprehensive drainage network for the Bharalu Basin to plan urban flood control. By collecting data including land use, rainfall, and floodwater levels, rainstorm runoffs were calculated and seven different trapezoidal drainage sections were designed to form a comprehensive urban drainage network [32]. In the study of flood risk management, most scholars established models and carried out researches based on hydrological data, and less considered the impact of social and economic factors.

Because of the lack of a sound allocation system, research on flood drainage rights is still in its infancy and has been applied only in areas highly prone to floods [33]. Yu et al. proposed that flood drainage rights refer to the rights to discharge waterlogged water caused by rainstorms and floods, then analyzed the basic characteristics and principles of flood drainage rights allocation [34]. Xu used ArcGIS to simulate the 2016 flood damages in the Qinhuai River Basin, proposed that the downstream cities could purchase flood drainage rights from the upstream region to reduce the flood control pressures, and formulated a pricing method for flood drainage rights trading [35]. Sun et al. formulated a framework of flood drainage rights allocation based on the pressure–state–response model and adopted an entropy-based matter–element model to evaluate the flood statuses and calculate the flood drainage rights of four cities in the Sunan Canal [36]. After that, they proposed a pricing model of flood drainage rights transactions based on the fair preference theory [37]. Referring to the initial allocation method of water and emission rights, Shen et al. constructed an initial allocation model of flood drainage rights based on a chaotic optimization–projection pursuit and selected Jiangsu Province in the Huai River Basin as the research object [38]. These studies only considered the allocation proportion of flood drainage rights, the transaction pricing based on the proportion, and did not involve the priority level of flood drainage rights in each place.

Flood drainage rights management is a multi-objective complex system [21]. Although multi-objective decision-making is applicable to the study of flood drainage rights allocation, the urgencies of flood drainage allocations in different regions of the basin are related not only to the natural environment but also to social factors. The uncertainties of the environment impede the implementation of multi-objective decision-making. In recent years, multi-objective decision-making based on fuzzy set theory has been widely used. Luu et al. used fuzzy sets to assess flood risks in Vietnam [39]. Goodarzi et al. developed a flood warning system based on atmospheric system prediction by using fuzzy TOPSIS multi-objective decision-making [40]. Kim et al. also used a fuzzy TOPSIS method to generate a flood disaster map of the Gam River in South Korea [41]. Song and Chung used the fuzzy TOPSIS method to evaluate the vulnerabilities of spatial floods to climate changes and rank seven cities in South Korea [42]. Intuitionistic fuzzy sets have been developed with the extension of fuzzy set theory, but some limitations on membership remained [43,44]. Then, Pythagorean fuzzy theory was proposed. This theory attracted the attention of many scholars. Yager and Abbasov proposed the concept of the Pythagorean fuzzy set (PFS), which extended the spatial ranges of membership and non-membership [45,46]. Zhang and Xu proposed the concept of Pythagorean fuzzy numbers (PFNs) and defined their operations, distances, score functions, precision functions, and similarities [47,48]. Peng and Yang defined their division and subtraction operations, then proposed the Pythagorean fuzzy superiority and inferiority ranking method to solve multi-criterion group decision-making problems [49]. There are many uncertainties in the management of flood drainage rights, such as the randomness of natural phenomena and the uncertainty caused by human activities. Using the Pythagorean fuzzy TOPSIS model can effectively solve the multi-criterion decision-making problem in the objective world. Considering the uncertainties of current regional flood drainage rights allocations and drainage demands in the Huai River Basin, our study considered regional social, economic, natural, and technical attributes, constructed a Pythagorean fuzzy TOPSIS model incorporating the urgencies of drainage rights allocations, and ranked the rights to provide a reference for flood risk management.

## 3. Materials and Methods

### 3.1. Research Area

One of the seven major river basins in China, the Huai River Basin is located at 111°55′–121°25′ E and 30°55′–36°36′ N, with a total area of about 27 × 10^4^ km^2^ in the eastern part of the country between the Yangtze and Yellow River Basins. The basin occupies the country’s North–South climatic transition zone. The regions north and south of the Huai River belong to the warm temperate and north subtropical zones, respectively. The average annual temperature ranges from 11 °C to 16 °C, with the highest and lowest monthly average temperatures at about 25 °C and 0 °C, respectively. The annual precipitation and average annual evaporation are about 920 mm and 900–1500 mm, respectively. Both precipitation and temperature show a trend of decline from south to north. Flood season is from June to September [50].

Under the influences of the concentrations of rainfall and inflow in the upper mountainous area, the water level crest of Hongze Lake in the lower reaches, and human activities, the Huai River Basin has the most frequent and severe flood disasters, with more than 350 floods having occurred in the past 2000 years [51]. According to statistics, a flood occurs every 2–3 years on average [52]. Since the beginning of the 21st century, regional floods have occurred in 2001, 2003, 2007, and 2009. During the 2017 flood season, heavy rainfall caused the main stream of the Huai River to exceed the warning water level. The current flood control situation in the basin is still severe.

**Figure 1:**
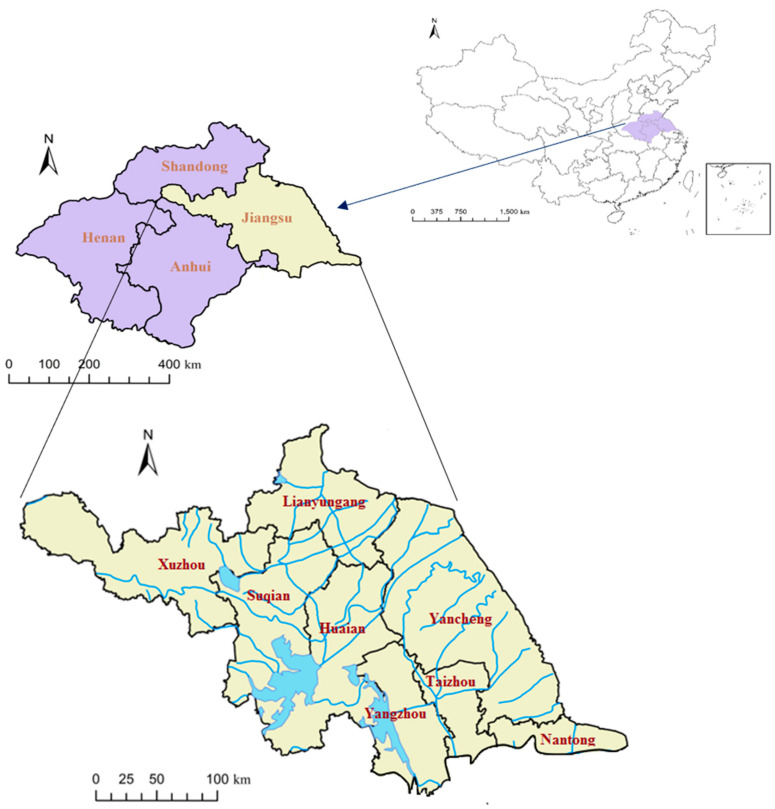
Location of Jiangsu Province in the Huai River Basin.

The Jiangsu Province in the Huai River Basin is in the lower reaches of the basin and includes Lianyungang, Xuzhou, Huaian, Suqian, Yangzhou, Taizhou, Yancheng, and Nantong. The area of Jiangsu Province in Huai River Basin is 6.53 × 10^4^ km^2^, accounting for 25% of the total area of Huai River Basin. The spatial and temporal distribution of rainfall and runoff varies greatly in Jiangsu Province, with obvious randomness and uncertainty. In terms of annual distribution, severe drought and little rain often occur in spring. The rainstorm occurs from June to September, with plum rain in the early stage (June to early July) and typhoon or thunderstorm in the later stage. During plum rain season, the rainfall range is wide in scope and the duration is long, which easily causes basin flood. Although the duration of typhoon rainstorm is short, the rainstorm is strong and destructive. Short-duration thunderstorms can cause local floods. The frequent floods in Jiangsu Province are mainly caused by two factors: the specific climate and underlying surface conditions; the insufficient scale of flood discharge project and the poor flow of flood into the Yangtze River. Figure 1 shows the location of Jiangsu Province in the Huai River Basin.

### 3.2. Index System of Flood Drainage Rights Allocation

“Pressure–State–Response” (PSR) framework is used to evaluate the impact of human activities on the ecological environment. PSR framework model can represent the interaction and sustainable development process between a system and external influencing factors. At present, the evaluation based on PSR framework has been widely used in ecological security, land use, mineral resources, transportation, economy, and other fields [53,54].

**Figure 2:**
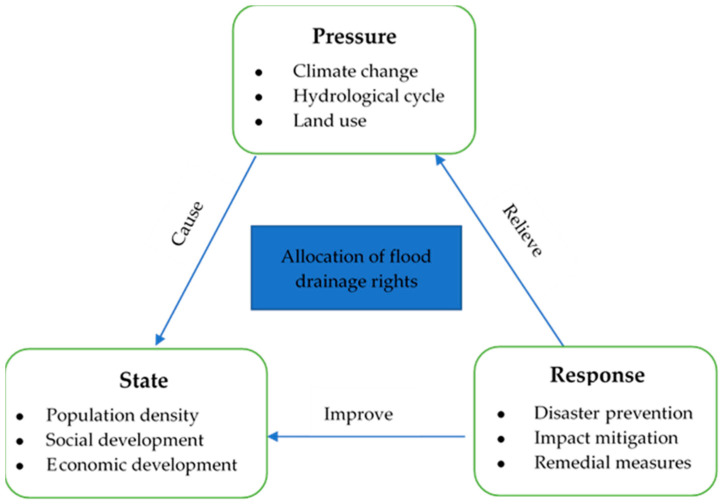
Pressure–state–response framework.

The urgency of the allocation of flood drainage rights is reflected not only in the threat of flood process to the city, but also in the ability of social and economic systems to prepare, respond, and recover. The management of flood drainage rights allocation should consider not only the spatial variabilities of regional topographic features, the locations of water resource management infrastructures, and the influences of different water resource regulation measures but also combine the watershed decision support system with the socio-economic system [55,56]. The dynamic process of flood drainage rights allocation conforms to the PSR framework. External factors (rain, etc.) exert pressure on the system and constitute stimulus inputs. State changes (such as damage to the downstream) occur in the system and their results are reflected in the adaptability of the system through some form of response (dealing with drainage conflicts). This study constructed an evaluation index system for ranking the urgency degrees of flood drainage rights allocations and analyzed the composition of the index system from the perspectives of pressure, state, and response (Figure 2).

(1) Pressure. The pressure index reflects the exposure of the system to external influencing factors, most of which are natural factors and are manifested as the loads imposed by flooding on the downstream areas. The higher the pressure load, the higher is the regional demand for the allocation of flood drainage rights. Climate change will aggravate the regional hydrological cycle [57]. Studies have shown that the rainfall will increase with increases in temperatures, leading to increased flood intensity [58]. Due to the well-developed water system in the lower reaches of the Huai River Basin, there is little difference in river network density among different regions. Flood drainage rights have greater demand in areas with abundant rainfall. Therefore, the rainfall deviation coefficient was selected to measure the effects of rainfall on the allocations of flood drainage rights. Because of the development of social economy and land use, as well as the aggravation of climate change, the frequency of flood disasters is increasing [59]. In addition, flood disasters will affect the production environment of agriculture and harm the growth of crops [60]. Crops are the main victims of natural disasters. Therefore, the density of cultivated land and the proportion of affected cropland were selected as indexes for the initial allocation of flood drainage rights.

(2) State. With the acceleration of urbanization, flood disasters will inevitably damage the social and economic systems of the region. Status indicators reflect the sensitivity of the system to external influencing factors and describe the changes in social and economic development caused by pressure loads. The higher the sensitivity, the more vulnerable the region is to the impact of floods. The sensitive factors include population, society, and economy. Studies have shown that higher population density is significantly correlated with water conflicts [61] and greater demand for flood drainage rights. Therefore, population density was selected to reflect the social situation of the region. Different levels of social development have different sensitivities to flood drainage rights. The spatial distribution difference index was selected as the social sensitivity evaluation index. The higher the level of economic development, the greater are the marginal losses caused by floods [38]. Therefore, the proportion of industrial added value in the GDP was selected to reflect the regional economic level.

(3) Response. When the capacities of urban drainage systems are limited, the floods caused by rainstorms will pose great threats to human production and life. The response index is the countermeasures taken by human beings facing such problems, i.e., the measures taken to prevent, reduce, restore, and remedy negative effects, which are manifested in adaptability to flooding. To realize the harmonious coexistence of “man” and “flood”, the river basin management department will increase investments in water conservancy projects, including reservoir dams, river channel restoration, and flood damages [62,63]. The investment contribution of drainage, sewage treatment rates, and density of drainage pipes in built-up areas were adopted as the measurement indicators of the drainage infrastructure capacity and contribution in each region.

### 3.3. Construction of Evaluation Model

**Figure 3:**
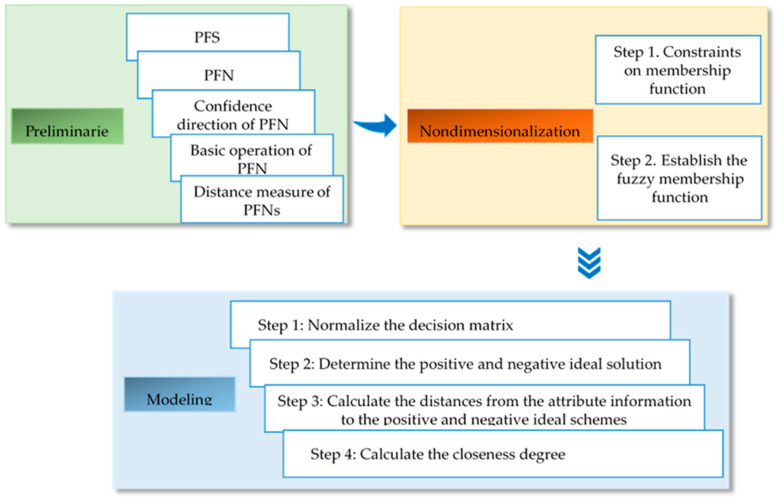
Structure of research method.

A PFS was proposed to solve the uncertainties of decision-making problems in the real world. As a generalization of the concept of fuzzy sets, intuitionistic fuzzy sets (IFSs), which have attracted the attention of many scholars, handle fuzzy information from the aspects of support, neutrality, and opposition. However, IFSs have certain limitations. The sum of the membership and non-membership degrees should not be greater than 1. A PFS compensates for this defect so that the decision-maker does not need to modify the intuitionistic fuzzy attribute values and can solve multi-criteria decision-making problems effectively. Figure 3 shows the structure of research method.

#### 3.3.1. Pythagoras Fuzzy Sets (PFSs)

In this study, the PFS multi-objective decision-making problem starts by approaching the ideal solution. The optimal alternative and worst schemes are obtained by fuzzy sets and the ranking is conducted according to the distance between the alternatives, the satisfaction standard with the optimal scheme, and the distance from the worst scheme.

**Definition** **1.** *Let set* X *be the universe and* $P=\left\{<x,\mu_{P}(x),v_{P}(x)>|x\in X\right\}$ *be the PFS on* X *,* *where* $\mu_{P}:X\to \left[0,1\right]$ *and* $v_{P}:X\to \left[0,1\right]$ *are the fuzzy sets on* X *,* *indicating that* x *belongs to the membership and non-membership degrees of* P *.* *All* x∈X *satisfy* $\mu_{P}^{2}(x)+v_{P}^{2}(x)\leq 1$ *.* $\pi_{P}(x)=\sqrt{1-\mu_{P}^{2}(x)-v_{P}^{2}(x)}$ *is the uncertainty of* x *’s belonging to* P *, which is called the hesitancy degree.* $<\mu_{P},v_{P}>$ *is a PFN, where* $\mu_{P},v_{P}\in \left[0,1\right]$ *and* $\mu_{P}^{2}(x)+v_{P}^{2}(x)\leq 1$ *.*

**Definition** **2.** *Let PFN* $P=<\mu_{P},v_{P}>$ *,* $r_{P}=\sqrt{\mu_{p}^{2}+v_{P}^{2}}$ *denote the confidence degree of* P *,* *and* $t_{P}=1-\frac{2}{\pi }\theta_{P}$ *denote the confidence direction of* P *,* *where* $\theta_{P}=arctan\frac{v_{P}}{\mu_{P}}\in \left[0,\frac{\pi }{2}\right]$ *.*

**Definition** **3.** *Let* $P_{1}=<\mu_{P_{1}},v_{P_{1}}>$ *and* $P_{2}=<\mu_{P_{2}},v_{P_{2}}>$ *be PFNs with the following definitions:* $P_{1}\geq P_{2}\Leftrightarrow \mu_{P_{1}}\geq \mu_{P_{2}}$ *,* $v_{\beta_{1}}\leq v_{\beta_{2}}$ *.*

**Definition** **4.** *Let PFN* $P=<\mu_{P},v_{P}>$ *. The score function is defined as* $S_{P}=\mu_{P}^{2}-v_{P}^{2}$ *,* $S_{P}\in \left[-1,1\right]$ *. Suppose* $P_{1}=<\mu_{P_{1}},v_{P_{1}}>$ *and* $P_{2}=<\mu_{P_{2}},v_{P_{2}}>\in PFNs$ *, then if* $S_{P_{1}}<S_{P_{2}}$ *, then* $P_{1}<P_{2}$ *. If* $S_{P_{1}}>S_{P_{2}}$ *, then* $P_{1}>P_{2}$ *, but if* $S_{P_{1}}=S_{P_{2}}$ *, then* $P_{1}\approx P_{2}$ *.*

**Definition** **5.** *According to Li et al. [64], PFNs are determined mainly by four factors or degrees: membership, non-membership, confidence, and direction of confidence. Let* $P_{1}=<\mu_{P_{1}},v_{P_{1}}>$ *and* $P_{2}=<\mu_{P_{2}},v_{P_{2}}>\in PFNs$ *, then the measure of the distances between the PFSs is as follows.*

*The Hamming distance of* $P_{1}$ *and* $P_{2}$ *is*

(1) $$d_{h}(P_{1},P_{2})=\frac{1}{4}(\left|\mu_{P_{1}}-\mu_{P_{2}}\right|+\left|v_{P_{1}}-v_{P_{2}}\right|+\left|r_{P_{1}}-r_{P_{2}}\right|+\left|t_{P_{1}}-t_{P_{2}}\right|)$$

*The Euclidean distance of* $P_{1}$ *and* $P_{2}$ *is*

(2) $$d_{e}(P_{1},P_{2})=\frac{1}{4}{\left[{\left(\mu_{P_{1}}-\mu_{P_{2}}\right)}^{2}+{\left(v_{P_{1}}-v_{P_{2}}\right)}^{2}+{\left(r_{P_{1}}-r_{P_{2}}\right)}^{2}+(t_{P_{1}}-t_{P_{2}}{)}^{2}\right]}^{\frac{1}{2}}$$

#### 3.3.2. Definition of Fuzzy Membership Degree of Each Factor Index

According to the definition of PFSs, the relationships among the membership function $\mu_{P}(x)$, non-membership function $v_{P}(x)$, and exponent $\pi_{P}(x)$ satisfy the following constraints:

(3) $$\mu_{P}^{2}(x)+v_{P}^{2}(x)+\pi_{P}^{2}(x)=1$$

So, $v_{P}^{2}(x)=\left[1-\pi_{P}^{2}(x)\right]-\mu_{P}^{2}(x)$ and $\delta_{P}^{2}(x)=1-\pi_{P}^{2}(x)$, then $v_{P}^{2}(x)=\delta_{P}^{2}(x)-\mu_{P}^{2}(x)$, where $\delta_{P}^{2}(x)$ is the index of non-hesitation. $0\leq \delta_{P}^{2}(x)\leq 1$ can be proved, so $\mu_{P}^{2}(x)+v_{P}^{2}(x)=\delta_{P}^{2}(x)$. The index can be assumed to be a constant value, i.e., $\pi_{P}^{2}(x)=a(0\leq a\leq 1)$. This assumption can be applied to most specific problems [65].

Because of the different evaluation indexes of flood drainage rights allocation, a unified comparison is impossible. Therefore, the evaluation values of each index are transformed into dimensionless values between (0,1) and the fuzzy membership function of each factor index is established.

(1) Rainfall deviation coefficient

Annual rainfall directly affects the ecological and hydrological processes, as well as the vegetation, of the basin [66]. Because of the differences in the rainfall of different regions, this index is quantified by the coefficient of rainfall deviation and its membership $\mu_{u_{1}}(t_{1})$ is defined as:

(4) $$\mu_{u_{1}}(t_{1})=\{\begin{array}{cc} 1-t_{1} & 0\leq t_{1}<1\\ 0 & t_{1}\geq 1 \end{array},\;t_{1}=\frac{R_{d}}{\bar{R}},$$

where $t_{1}$ is the rainfall deviation coefficient, $R_{d}$ is the rainfall data of the current year, and $\bar{R}$ is the average rainfall over the years.

(2) Cultivated land density

The membership degree of cultivated land density can be determined by

(5) $$\mu_{u_{2}}(t_{2})=t_{2},\;t_{2}=\frac{L_{c}}{L},$$

where $t_{2}$ is the density of cultivated land, $L_{c}$ is the cultivated area, and L is the land area.

(3) Proportion of affected cropland

The membership degree of the proportion of affected cropland can be determined by

(6) $$\mu_{u_{3}}(t_{3})=t_{3},\;t_{3}=\frac{L_{a}}{L},$$

where $t_{3}$ is the proportion of affected cropland, $L_{a}$ is the area of affected cropland, and L is the total land area.

(4) Population density

The membership degree of population density can be determined by

(7) $$\mu_{u_{7}}(t_{7})=t_{7},\;t_{7}=\frac{P_{r}}{L},$$

where $t_{7}$ is the population density, $P_{r}$ is the resident population, and L is the total land area.

(5) Spatial distribution difference index

The membership degree of the spatial distribution difference index can be determined by

(8) $$\mu_{u_{8}}(t_{8})=\{\begin{array}{l} \begin{array}{cc} 0.9\begin{array}{cc} & t_{8}\geq 2.1 \end{array} & \end{array}\\ \begin{array}{cc} 0.7 & 2.0\leq t_{8}<2.1 \end{array}\\ \begin{array}{l} \begin{array}{cc} 0.5 & 1.9\leq t_{8}<2.0 \end{array}\\ \begin{array}{cc} 0.3 & 1.8\leq t_{8}<1.8 \end{array} \end{array}\\ \begin{array}{cc} 0.1\begin{array}{cc} & t_{8}<1.8 \end{array} & \end{array} \end{array},\;t_{8}=\frac{I_{u}}{I_{r}},$$

where $t_{8}$ is the spatial distribution difference index, $I_{u}$ is the per capita disposable income of urban residents, and $I_{r}$ is the per capita disposable income of rural residents.

(6) Proportion of industrial added value in GDP

The membership degree of the proportion of industrial added value in GDP can be determined by

(9) $$\mu_{u_{9}}(t_{9})=t_{9},$$

where $t_{9}$ is the proportion of industrial added value in the GDP.

(7) Investment contribution of drainage

The membership degree of the investment contribution of drainage can be determined by

(10) $$\mu_{u_{4}}(t_{4})=t_{4},\;t_{4}=\frac{I_{r}}{I},$$

where $t_{4}$ is the investment contribution of drainage, $I_{r}$ is the investments of regional water conservancy project constructions, and I is the total investments in the basin.

(8) Sewage treatment rate

The membership degree of the sewage treatment rate can be determined by

(11) $$\mu_{u_{5}}(t_{5})=t_{5},$$

where $t_{5}$ is the sewage treatment rate.

(9) Density of drainage pipes in built-up areas

The membership degree of the drainage pipe density in built-up areas can be determined by

(12) $$\mu_{u_{6}}(t_{6})=\{\begin{array}{l} \begin{array}{cc} 0.9\begin{array}{cc} & t_{6}\geq 16 \end{array} & \end{array}\\ \begin{array}{cc} 0.7 & 14\leq t_{6}<16 \end{array}\\ \begin{array}{l} \begin{array}{cc} 0.5 & 12\leq t_{6}<14 \end{array}\\ \begin{array}{cc} 0.3 & 10\leq t_{6}<12 \end{array} \end{array}\\ \begin{array}{cc} 0.1\begin{array}{cc} & t_{6}<10 \end{array} & \end{array} \end{array},\;t_{6}=\frac{P_{d}}{L_{u}},$$

where $t_{6}$ is the density of drainage pipes in built-up areas, $P_{d}$ is the length of urban drainage pipes, and $L_{u}$ is the size of the built-up area.

#### 3.3.3. Pythagoras Fuzzy TOPSIS Evaluation Model

The Pythagorean multi-objective decision-making problem can be expressed as follows. Let $\left\{H_{1},H_{2},\cdots ,H_{m}\right\}(m\geq 2)$ be the feasible scheme set and $\left\{u_{1},u_{2},\cdots ,u_{n}\right\}$ be the attribute set. These sets are the influencing factors of flood drainage rights allocation. The weight of each influencing factor is $W=(w_{1},w_{2},\cdots ,w_{n})$, which satisfies $0\leq w_{j}\leq 1$ and $\sum_{j=1}^{n}w_{j}=1$.

The evaluation value $A_{ij}=<\mu_{ij},v_{ij}>$ of scheme $H_{i}$ under attribute $u_{j}$ is a PFN, i=1,2,⋯,m, j=1,2,⋯,n. The Pythagorean fuzzy decision matrix is ${\left(A_{ij}\right)}_{m\times n}$, which can be expressed as

(13) $${\left(A_{ij}\right)}_{m\times n}=\left(\begin{array}{cccc} <\mu_{11},v_{11}> & <\mu_{12},v_{12}> & \cdots & <\mu_{1n},v_{1n}>\\ <\mu_{21},v_{21}> & <\mu_{22},v_{22}> & \cdots & <\mu_{2n},v_{2n}>\\ \vdots & \vdots & \vdots & \vdots \\ <\mu_{m1},v_{m1}> & <\mu_{m2},v_{m2}> & \cdots & <\mu_{mn},v_{mn}> \end{array}\right)$$

Step 1: Normalize the decision matrix.

To eliminate the influences of different types of attributes, the normalization of ${\left(A_{ij}\right)}_{m\times n}$ is necessary. If the normalized decision matrix is ${\left({\tilde{A}}_{ij}\right)}_{m\times n}$ and ${\tilde{A}}_{ij}=<{\tilde{\mu }}_{ij},{\tilde{v}}_{ij}>$, then ${\tilde{\mu }}_{ij}=\mu_{ij}$ and ${\tilde{v}}_{ij}=v_{ij}$ denote the benefit attributes while ${\tilde{\mu }}_{ij}=v_{ij}$ and ${\tilde{v}}_{ij}=\mu_{ij}$ denote the cost attributes.

Step 2: Determine the positive and negative ideal solution.

The TOPSIS method is extended to PFNs to find the optimal solution from the positive $H^{+}$ and negative $H^{-}$ ideal solutions, i.e., fractional functions combined with PFNs, which indicate that the score function of the PFNs is the highest or lowest, respectively, and satisfies Definitions 3 and 4:

(14) $$H^{+}=\left\{A_{1}^{+},A_{2}^{+},\cdots ,A_{n}^{+}\right\},\;A_{j}^{+}=(\mu_{j}^{+},v_{j}^{+}),\;\mu_{j}^{+}=\underset{1\leq i\leq m}{max}{\tilde{\mu }}_{ij},\;v_{j}^{+}=\underset{1\leq i\leq m}{min}{\tilde{v}}_{ij}$$

(15) $$H^{-}=\left\{A_{1}^{-},A_{2}^{-},\cdots ,A_{n}^{-}\right\},\;A_{j}^{-}=(\mu_{j}^{-},v_{j}^{-}),\;\mu_{j}^{-}=\underset{1\leq i\leq m}{min}{\tilde{\mu }}_{ij},\;v_{j}^{-}=\underset{1\leq i\leq m}{max}{\tilde{v}}_{ij}$$

Step 3: Calculate the distances from the attribute information of each scheme to the positive and negative ideal schemes.

The Hamming or Euclidean distance is used to calculate the distances $d({\tilde{A}}_{ij},A_{j}^{+})$ and $d({\tilde{A}}_{ij},A_{j}^{-})$ from the attribute information of each scheme $H_{i}$ to the positive and negative ideal schemes, i=1,2,⋯,m and j=1,2,⋯,n:

(16) $$D(H_{i},H^{+})=\sum_{j=1}^{n}w_{j}d({\tilde{A}}_{ij},A_{j}^{+}),\;D_{min}(H_{i},H^{+})=\underset{1\leq i\leq m}{min}D(H_{i},H^{+})$$

(17) $$D(H_{i},H^{-})=\sum_{j=1}^{n}w_{j}d({\tilde{A}}_{ij},A_{j}^{-}),\;D_{max}(H_{i},H^{-})=\underset{1\leq i\leq m}{max}D(H_{i},H^{-})$$

The shorter or longer the distance to the positive or negative ideal solution, respectively, the more closely the scheme meets the standard.

Step 4: Calculate the closeness degree.

The distances can be ranked by modifying the closeness degree $\zeta (H_{i})$, $\zeta (H_{i})\leq 0(i=1,2,\cdots ,m)$. A larger $\zeta (H_{i})$ indicates that $H_{i}$ is closer to the optimal result:

(18) $$\zeta (H_{i})=\frac{D(H_{i},H^{-})}{D_{max}(H_{i},H^{-})}-\frac{D(H_{i},H^{+})}{D_{min}(H_{i},H^{+})}$$

## 4. Results and Discussions

### 4.1. Data Sources and Determination of Index Weights

For Jiangsu Province, the urgencies of the allocations of flood drainage rights for Xuzhou ($H_{1}$), Nantong ($H_{2}$), Lianyungang ($H_{3}$), Huaian ($H_{4}$), Yancheng ($H_{5}$), Yangzhou ($H_{6}$), Taizhou ($H_{7}$), and Suqian ($H_{8}$) were ranked. Nantong, Taizhou, and Yangzhou belong to the central area of Jiangsu Province, and Huaian, Suqian, Yancheng, Xuzhou, and Lianyungang belong to the northern areas of Jiangsu Province.

To ensure the accuracy of the data, the index data were taken from the Huai River Water Resources Bulletin, Jiangsu Statistical Yearbook, and Jiangsu Water Conservancy Yearbook. Some indicators (such as rainfall deviation coefficient, spatial distribution, and difference index) were calculated using the statistical data (Table 1).

From the perspective of pressure layer, there are obvious differences in rainfall among cities. Yancheng and Nantong are coastal areas which are significantly affected by monsoon precipitation and the plum rain. The rainfall deviation is relatively large, and Yancheng and Nantong are threatened by flood disasters. The land area of northern Jiangsu is more extensive than that in central Jiangsu. Xuzhou, Suqian, and Lianyungang have higher density of cultivated land, while Nantong has the lowest density. Affected by the factors such as climate, the disaster area of crops in central areas of Jiangsu accounts for a large proportion.

From the perspective of state layer, Taizhou has the highest population density, followed by Xuzhou, and Yancheng has the lowest population density. The spatial distribution difference index of each city is relatively average. The proportion of industrial added value in GDP of Yangzhou and Taizhou is higher, and Huaian has the lowest proportion.

From the perspective of response layer, Yancheng has the highest investment contribution, accounting for more than 30% of all regions, and Taizhou has the lowest contribution. The sewage treatment rate is high in all regions. The density of drainage pipeline is highest in Nantong, followed by Huaian, Xuzhou is the lowest.

The lower the correlation between different indicators, that is, the higher the degree of conflict between indicators, the more useful information can be provided. The improved entropy weight method with a conflict coefficient [20] was used to determine the weights of the above factors affecting the allocations, as shown in Table 2. According to the result of index weight, the index weight of pressure layer is the highest, followed by that of the state layer, and the index weight of response layer is the lowest. The weight of drainage pipe density in the built-up area is the highest, and the weight of sewage treatment rate is the lowest.

### 4.2. Ranking of Urgency Degrees of Flood Drainage Rights Allocations

By the definition of membership degree $v_{P}^{2}(x)=\delta_{P}^{2}(x)-\mu_{P}^{2}(x)$ and acquisition of the index data, the Pythagorean fuzzy decision matrix ${\left(A_{ij}\right)}_{m\times n}$ was established.

(1) The fuzzy decision matrix was normalized. Since the attribute criteria included both benefit and cost types, normalization was necessary to obtain Table 2.

(2) The positive and negative ideal schemes were determined. Equations (14) and (15) were used to obtain the positive $H^{+}$ and negative $H^{-}$ ideal schemes, respectively. The results are also shown in Table 3.

(3) The distances from the attribute information of each scheme to the positive and negative ideal schemes were calculated by using Definition 5. The Hamming distance was selected, and the distance matrices in Table 4 and Table 5 were obtained.

(4) The relative closeness degree of each scheme was calculated. Combined with the weight vector of each attribute, the relative closeness degree of scheme S was calculated by Equation (18). The results are shown in Table 6. The ranking of the urgencies is Nantong, Taizhou, Huaian, Yangzhou, Suqian, Yancheng, Xuzhou, and Lianyungang.

Taking into consideration that different distance definitions and measurement methods of PFN sets influence the ranking results, this study calculated the ranking of the urgencies under different measurement methods. Table 7 shows the results calculated by the use of the Hamming distance, Euclidean distance, and methods of Chen, Y. and Yu, J. [61] respectively. The results obtained by all three methods are completely consistent and further prove the validity of the ranking.

### 4.3. Evaluation of Results

**Figure 4:**
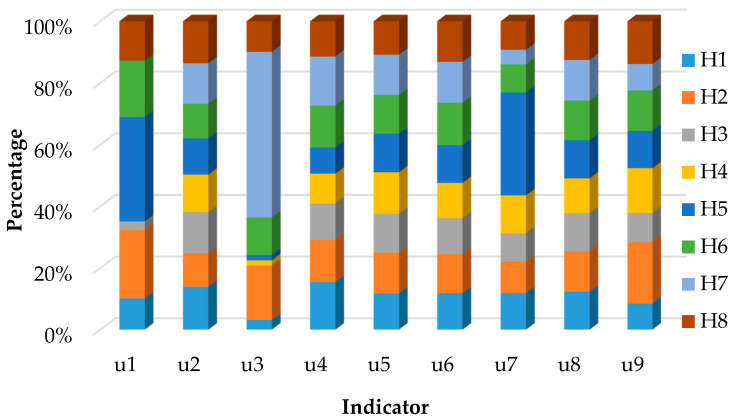
Proportions of indicators in eight regions.

The results of the ranking showed the following:

(1) Flood drainage rights management is a complex system. There are many factors affecting the allocation of flood drainage rights, including geography, environment, disaster mechanism, and so forth. The main factors are the natural system, social economy system, and the combination of the two aspects. The ranking of the urgency degrees of flood drainage rights allocation was the result of the comprehensive consideration of system pressure, state, response, and other factors. For Jiangsu Province, pressure layer played a dominant role in the allocation and strongly affected the ranking.

(2) Nantong had the highest urgency, followed by Taizhou, and Lianyungang’s ranking last. Combined with the original index data of each region, Nantong had obvious advantages in each attribute value. Nantong has a dense population, high level of urbanization, and high spatial distribution difference index. In recent years, the cultivated land in Nantong has decreased rapidly, the soil erosion has been aggravated, and the annual rainfall has shown an obvious upward trend, which increases the affected cropland and indicates that Nantong is more likely to be affected by flooding. The local government has strengthened the water conservancy infrastructure and focused on the treatment of water pollution. The sewage treatment rate and drainage pipeline density in the built-up areas of Nantong are the highest among those of the eight regions (Figure 4). Therefore, Nantong ranks first in the allocation of flood drainage rights. However, the indicators of the pressure, state, and response layers of Lianyungang were at the lowest levels.

(3) The urgency of flood drainage rights allocation in the central areas, such as Nantong, Taizhou, and Yangzhou, of Jiangsu were higher than in the northern areas, such as Huaian, Suqian, Yancheng, Xuzhou, and Lianyungang. Regarding the natural factors, Jiangsu Province belongs to the transitional zone between the temperate and subtropical zones. Under the influence of the El Niño phenomenon, floods often occur as a result of the excessive rainfall. In addition, most of the area is located in the plain area, the terrain elevation is low and the terrain variation is small, which aggravates the extent of the flood damage. Average rainfall deviation and average proportion of affected cropland coefficient were also higher and led to the higher urgency in the central areas. Moreover, the higher sewage treatment rate and drainage pipe density in the built-up areas in the central areas also indicated higher demand for improving the capacity and quality of the drainage systems.

Regarding the social and economic development factors, the difference in the levels of the social and economic development of the central and northern areas formed a gap in the sensitivities and responses to flooding. The average population density, spatial distribution difference index, and proportion of industrial added value in the GDP in the central areas were also higher. Because of the rapid population growth and pace of urbanization in the central areas, the flood storage and regulation functions of the river channels have been severely weakened and the drainage of rivers has become difficult. When flood disasters occur in areas with higher levels of social and economic development, greater damages are suffered and the recovery costs are higher; hence, the urgency of the allocations of flood drainage rights in central Jiangsu ranked higher.

The allocation management of flood drainage rights mainly analyzes the extent to which human social and economic systems are affected by flood, as well as the capacity of social and economic systems to prevent, reduce, and recover from the impact of natural disasters [67]. From the perspective of regional sustainable development, improving the ability to respond to and recover from flood disasters can greatly reduce the social and economic losses when flood disasters occur. The higher the level of social and economic development of the city, the greater the impact of the flood and the higher the recovery cost. The economic strength of the city can also effectively reduce the impact caused of disasters, such as accelerating the construction of regional road network and enhancing regional evacuation capacity [68]. Good ecological environment can buffer the flood. The urbanization process has changed the underlying surface conditions and the runoff conditions [69]. Therefore, it is necessary to accelerate the construction of forest vegetation and the control of soil erosion, and improve the soil’s capacity of water storage and microclimate regulation.

From the perspective of integrated flood management in river basins, it is suggested to explore the causes of climate dynamics that lead to rainfall changes and summarize their occurrence rules, so as to provide guidance for the formulation of flood control management strategies. At the same time, the level of long-term rainfall forecast can be improved and a basis provided for timely response to flood. On this basis, flood control scheduling schemes of the river basin are formulated to alleviate drainage contradictions among different regions, reduce the overall loss of the river basin, and ensure the sustainable economic, social, and ecological development of the basin.

## 5. Conclusions

Since the allocation process of flood drainage rights is a multi-objective complex system with uncertainty, this study aimed to rank the priorities of flood drainage rights allocations by using the natural conditions, technical management, and the levels of social and economic development of the areas in a river basin as relevant factors to construct an index system. Jiangsu Province in the Huai River Basin of China was taken as the research object, and the Pythagoras fuzzy TOPSIS method was used to analyze the urgencies of allocations. Given that safe displacement could not meet the drainage demand, the priority of allocation was Nantong, Taizhou, Huaian, Yangzhou, Suqian, Yancheng, Xuzhou, and Lianyungang. Finally, the validity of this study was verified by a comparison of rankings obtained under different measurement methods. This study is conducive to the formulation of drainage rights distribution policies by the river basin administrative departments, to alleviate the drainage contradictions among different administrative regions in the basin, to reduce the overall loss of the river basin, and to maintain the stable development.

The contributions of this study are as follows: (1) With the dynamic processes of watershed decision support and social economic systems, the uncertainty of flood drainage rights allocation was analyzed from the perspectives of the pressure, state, and response layers and an index system of flood drainage rights allocation was proposed; (2) The PFS and TOPSIS methods were combined to solve multi-objective decision-making problems in flood drainage rights allocation.

The urgency ranking of flood drainage rights allocation in a region proposed by this study can be calculated by using the natural, technical, social, and economic data of the region to provide a reference for other basins and regions. Some aspects of this study could be improved: (1) The distribution of drainage rights involves natural, economic, and social factors. Because of the wide regional differences in different watersheds, the allocation index system can be further optimized by combining with the actual situation of each basin; (2) The urgencies can be combined with the initial proportion of allocation and the transaction price of drainage rights to realize a harmonious allocation of drainage rights in the basin from the perspectives of space and time.

## Figures and Tables

**Table 1:** Values of indicators.

Factor Layer	Index Layer	$H_{1}$	$H_{2}$	$H_{3}$	$H_{4}$	$H_{5}$	$H_{6}$	$H_{7}$	$H_{8}$
Pressure (P)	Rainfall deviation coefficient ($u_{1}$)	0.07	0.14	0.02	0.00	0.22	0.12	0.00	0.08
	Cultivated land density ($u_{2}$)	0.54	0.42	0.51	0.47	0.46	0.43	0.51	0.53
	Proportion of affected cropland ($u_{3}$)	0.02	0.09	0.00	0.01	0.01	0.06	0.27	0.05
State (S)	Population density ($u_{4}$)—10^3^ person/km^2^	0.78	0.69	0.59	0.49	0.43	0.68	0.80	0.58
	Spatial distribution difference index ($u_{5}$)	1.86	2.09	1.98	2.11	1.97	1.97	2.05	1.71
	Proportion of industrial added value in GDP ($u_{6}$)	0.37	0.39	0.36	0.36	0.38	0.43	0.41	0.41
Response (R)	Investment contribution of drainage ($u_{7}$)	0.12	0.10	0.09	0.12	0.33	0.09	0.05	0.09
	Sewage treatment rate ($u_{8}$)	0.89	0.94	0.89	0.81	0.89	0.93	0.94	0.90
	Density of drainage pipe in built-up areas ($u_{9}$)—km/km^2^	8.87	20.69	9.90	15.06	12.61	13.64	8.90	14.44

**Table 2:** Index weight of each influencing factor.

Factor Layer	Factor Layer Weight	Index Layer	Index Weight
P	0.3458	$u_{1}$	0.0953
		$u_{2}$	0.1247
		$u_{3}$	0.1258
S	0.3310	$u_{4}$	0.1014
		$u_{5}$	0.1069
		$u_{6}$	0.1227
R	0.3232	$u_{7}$	0.1052
		$u_{8}$	0.0808
		$u_{9}$	0.1373

**Table 3:** Normalized Pythagorean fuzzy decision matrix.

Scheme	$u_{1}$	$u_{2}$	$u_{3}$	$u_{4}$	$u_{5}$	$u_{6}$	$u_{7}$	$u_{8}$	$u_{9}$
$H_{1}$	<0.95,0.07>	<0.54,0.78>	<0.02,0.95>	<0.78,0.54>	<0.30,0.90>	<0.37,0.87>	<0.12,0.94>	<0.89,0.33>	<0.10,0.94>
$H_{2}$	<0.94,0.14>	<0.42,0.85>	<0.09,0.94>	<0.69,0.65>	<0.70,0.64>	<0.39,0.86>	<0.10,0.94>	<0.94,0.12>	<0.90,0.30>
$H_{3}$	<0.95,0.02>	<0.51,0.80>	<0.00,0.95>	<0.59,0.74>	<0.50,0.81>	<0.36,0.88>	<0.09,0.94>	<0.89,0.34>	<0.10,0.94>
$H_{4}$	<0.95,0.00>	<0.47,0.82>	<0.01,0.95>	<0.49,0.81>	<0.90,0.30>	<0.36,0.88>	<0.12,0.94>	<0.81,0.49>	<0.70,0.64>
$H_{5}$	<0.92,0.22>	<0.46,0.83>	<0.01,0.95>	<0.43,0.85>	<0.50,0.81>	<0.38,0.87>	<0.33,0.89>	<0.89,0.33>	<0.50,0.81>
$H_{6}$	<0.94,0.12>	<0.43,0.84>	<0.06,0.95>	<0.68,0.66>	<0.50,0.81>	<0.43,0.85>	<0.09,0.94>	<0.93,0.20>	<0.50,0.81>
$H_{7}$	<0.95,0.00>	<0.51,0.80>	<0.27,0.91>	<0.80,0.50>	<0.90,0.30>	<0.41,0.85>	<0.05,0.95>	<0.94,0.12>	<0.10,0.94>
$H_{8}$	<0.95,0.08>	<0.53,0.79>	<0.05,0.95>	<0.58,0.75>	<0.10,0.94>	<0.41,0.85>	<0.09,0.94>	<0.90,0.29>	<0.70,0.64>
$H^{+}$	<0.95,0.00>	<0.54,0.78>	<0.27,0.91>	<0.80,0.50>	<0.90,0.30>	<0.43,0.85>	<0.33,0.89>	<0.94,0.12>	<0.90,0.30>
$H^{-}$	<0.92,0.22>	<0.42,0.85>	<0.00,0.95>	<0.43,0.85>	<0.10,0.94>	<0.36,0.88>	<0.05,0.95>	<0.81,0.49>	<0.10,0.94>

**Table 4:** Distance from attribute information of each scheme to $H^{+}$.

Scheme	$u_{1}$	$u_{2}$	$u_{3}$	$u_{4}$	$u_{5}$	$u_{6}$	$u_{7}$	$u_{8}$	$u_{9}$
$H_{1}$	0.0280	0.0000	0.1152	0.0237	0.4476	0.0319	0.1042	0.1029	0.5428
$H_{2}$	0.0629	0.0707	0.0840	0.0946	0.2018	0.0196	0.1113	0.0000	0.0000
$H_{3}$	0.0075	0.0164	0.1216	0.1651	0.3370	0.0360	0.1168	0.1079	0.5428
$H_{4}$	0.0000	0.0419	0.1184	0.2301	0.0000	0.0394	0.1026	0.1933	0.2018
$H_{5}$	0.0982	0.0508	0.1181	0.2662	0.3370	0.0255	0.0000	0.1011	0.3370
$H_{6}$	0.0513	0.0636	0.0959	0.1011	0.3370	0.0000	0.1166	0.0358	0.3370
$H_{7}$	0.0000	0.0188	0.0000	0.0000	0.0000	0.0093	0.1359	0.0004	0.5428
$H_{8}$	0.0355	0.0082	0.1007	0.1761	0.5428	0.0098	0.1161	0.0799	0.2018

**Table 5:** Distance from attribute information of each scheme to $H^{-}$.

Scheme	$u_{1}$	$u_{2}$	$u_{3}$	$u_{4}$	$u_{5}$	$u_{6}$	$u_{7}$	$u_{8}$	$u_{9}$
$H_{1}$	0.0702	0.0707	0.0064	0.2425	0.0953	0.0074	0.0317	0.0904	0.0000
$H_{2}$	0.0353	0.0000	0.0376	0.1715	0.3410	0.0198	0.0245	0.1933	0.5428
$H_{3}$	0.0907	0.0543	0.0000	0.1011	0.2058	0.0034	0.0190	0.0854	0.0000
$H_{4}$	0.0982	0.0287	0.0032	0.0361	0.5428	0.0000	0.0333	0.0000	0.3410
$H_{5}$	0.0000	0.0199	0.0035	0.0000	0.2058	0.0138	0.1359	0.0922	0.2058
$H_{6}$	0.0469	0.0071	0.0257	0.1651	0.2058	0.0394	0.0192	0.1576	0.2058
$H_{7}$	0.0982	0.0519	0.1216	0.2662	0.5428	0.0300	0.0000	0.1930	0.0000
$H_{8}$	0.0627	0.0624	0.0209	0.0901	0.0000	0.0296	0.0197	0.1135	0.3410

**Table 6:** Decision results of Pythagoras fuzzy TOPSIS method.

Scheme	$D(H_{i},H^{+})$	$D(H_{i},H^{-})$	$\zeta (H_{i})$	Priority Order
$H_{1}$	0.1615	0.0626	−1.9385	7
$H_{2}$	0.0707	0.1571	0.0000	1
$H_{3}$	0.1708	0.0570	−2.0540	8
$H_{4}$	0.1024	0.1254	−0.6511	3
$H_{5}$	0.1511	0.0766	−1.6514	6
$H_{6}$	0.1326	0.0952	−1.2705	4
$H_{7}$	0.0923	0.1354	−0.4447	2
$H_{8}$	0.1405	0.0872	−1.4340	5

**Table 7:** Rankings obtained by three measurement methods.

Measurement Methods	Results of Modified Closeness Degree $\zeta (H_{i})$ of Each Scheme	Rankings							
		$H_{1}$	$H_{2}$	$H_{3}$	$H_{4}$	$H_{5}$	$H_{6}$	$H_{7}$	$H_{8}$
Hamming distance	(−1.9385, 0.0000, −2.0540, −0.6511, −1.6514, −1.2705, −0.4447, −1.4340)	7	1	8	3	6	4	2	5
Euclidean distance	(−1.8531, 0.0000, −1.9629, −0.6303, −1.5669, −1.1985, −0.3823, −1.3734)	7	1	8	3	6	4	2	5
Methods of Chen, Y. and Yu, J.	(−2.1182, 0.0000, −2.3322, −0.7702, −2.0549, −1.5911, −0.5911, −1.5911)	7	1	8	3	6	4	2	5

## References

[1] Alfieri L., Bisselink B., Dottori F., Naumann G., de Roo A., Salamon P., Wyser K., Feyen L. (2017). Global projections of river flood risk in a warmer world. Earths Future.

[2] Trenberth K. (2007). Climate change—Warmer oceans, stronger hurricanes. Sci. Am..

[3] Allamano P., Claps P., Laio F. (2009). Global warming increases flood risk in mountainous areas. Geophys. Res. Lett..

[4] Milly P., Wetherald R.T., Dunne K.A., Delworth T.L. (2002). Increasing risk of great floods in a changing climate. Nature.

[5] Jongman B., Hochrainer-Stigler S., Feyen L., Aerts J.C.J.H., Mechler R., Botzen W.J.W., Bouwer L.M., Pflug G., Rojas R., Ward P.J. (2014). Increasing stress on disaster-risk finance due to large floods. Nat. Clim. Chang..

[6] Mishra A.K., Meer M.S., Nagaraju V. (2019). Satellite-based monitoring of recent heavy flooding over north-eastern states of India in July 2019. Nat. Hazards.

[7] Sharma S.K., Kwak Y., Kumar R., Sarma B. (2018). Analysis of Hydrological Sensitivity for Flood Risk Assessment. ISPRS Int. J. Geo-Inf..

[8] Islam M.M., Al Mamun M.A. (2020). Beyond the risks to food availability—Linking climatic hazard vulnerability with the food access of delta-dwelling households. Food Secur..

[9] Lopez P.L., Sultana T., Kafi M.A.H., Hossain M.S., Khan A.S., Masud M.S. (2020). Evaluation of Global Water Resources Reanalysis Data for Estimating Flood Events in the Brahmaputra River Basin. Water Resour. Manag..

[10] Cirillo G., Albrecht E. (2015). The importance of law in flood risk management. WIT Transactions on Ecology and the Environment.

[11] Marks D., Connell J., Ferrara F. (2020). Contested notions of disaster justice during the 2011 Bangkok floods: Unequal risk, unrest and claims to the city. Asia Pac. Viewp..

[12] Singkran N., Kandasamy J. (2016). Developing a strategic flood risk management framework for Bangkok, Thailand. Nat. Hazards.

[13] Ng S. (2016). Governance beyond the government: Responding to a reactionary flood governance regime in Ayutthaya, Thailand. Habitat Int..

[14] Kharel G., Kirilenko A. (2018). Comparing CMIP-3 and CMIP-5 climate projections on flooding estimation of Devils Lake of North Dakota, USA. PeerJ.

[15] Ma J., Hipel K.W., De M. (2011). Devils lake emergency outlet diversion conflict. J. Environ. Manag..

[16] Wilby R.L., Keenan R. (2012). Adapting to flood risk under climate change. Prog. Phys. Geogr. Earth Environ..

[17] Grabs W., Tyagi A.C., Hyodo M. (2007). Integrated flood management. Water Sci. Technol..

[18] Qin H., Zhou J., Lu Y., Li Y., Zhang Y. (2010). Multi-objective Cultured Differential Evolution for Generating Optimal Trade-offs in Reservoir Flood Control Operation. Water Resour. Manag..

[19] Yu F., Wang Y., Yuan X., Jiang S. (2014). Preliminary study on concept and its basic characteristics of flood drainage right. J. Irrig. Drain..

[20] Zhang D., Shen J., Sun F., Liu B., Wang Z., Zhang K., Li L. (2019). Research on the Allocation of Flood Drainage Rights of the Sunan Canal Based on a Bi-level Multi-Objective Programming Model. Water.

[21] Zhang D., Shen J., Liu P., Zhang Q., Sun F. (2020). Use of Fuzzy Analytic Hierarchy Process and Environmental Gini Coefficient for Allocation of Regional Flood Drainage Rights. Int. J. Environ. Res. Public Health.

[22] Winz I., Brierley G., Trowsdale S. (2009). The Use of System Dynamics Simulation in Water Resources Management. Water Resour. Manag..

[23] Dutta D., Herath S., Musiakec K. (2003). A mathematical model for flood loss estimation. J. Hydrol..

[24] De Brito M.M., Evers M. (2016). Multi-criteria decision-making for flood risk management: A survey of the current state of the art. Nat. Hazards Earth Syst..

[25] Merz B., Hall J., Disse M., Schumann A. (2010). Fluvial flood risk management in a changing world. Nat. Hazards Earth Syst..

[26] Jongman B., Kreibich H., Apel H., Barredo J.I., Bates P.D., Feyen L., Gericke A., Neal J., Aerts J.C.J.H., Ward P.J. (2012). Comparative flood damage model assessment: Towards a European approach. Nat. Hazards Earth Syst..

[27] Hazarika N., Barman D., Das A.K., Sarma A.K., Borah S.B. (2018). Assessing and mapping flood hazard, vulnerability and risk in the Upper Brahmaputra River valley using stakeholders’ knowledge and multicriteria evaluation (MCE). J. Flood Risk Manag..

[28] Lee G., Jun K.S., Chung E.S. (2015). Group decision-making approach for flood vulnerability identification using the fuzzy VIKOR method. Nat. Hazards Earth Syst..

[29] Tehrany M.S., Pradhan B., Jebur M.N. (2013). Spatial prediction of flood susceptible areas using rule based decision tree (DT) and a novel ensemble bivariate and multivariate statistical models in GIS. J. Hydrol..

[30] Sikder M.S., David C.H., Allen G.H., Qiao X., Nelson E.J., Matin M.A. (2019). Evaluation of Available Global Runoff Datasets Through a River Model in Support of Transboundary Water Management in South and Southeast Asia. Front. Environ. Sci..

[31] Wang X., Wang S., Cui H. (2019). Application of the Entropy Spectral Method for Streamflow and Flood-Affected Area Forecasting in the Brahmaputra River Basin. Entropy.

[32] Sarmah T., Das S. (2018). Urban flood mitigation planning for Guwahati: A case of Bharalu basin. J. Environ. Manag..

[33] Zhang J., Zhang C., Liu L., Shen J., Zhang D., Sun F. (2019). Necessity and feasibility of allocation and trading of drainage rights in Jiangsu Province. Water Resour. Prot..

[34] Yu F., Wang Y., Yuan X. (2013). Preliminary study on the reasonable allocation of flood drainage right for midstream of Huaihe River. Appl. Mech. Mater..

[35] Xu X. (2019). Spatial Allocation of Flood Drainage Right Based on the Compensation Mechanism of Detention Basin. Master’s Thesis.

[36] Sun F., Lai X., Shen J., Nie L., Gao X. (2020). Initial allocation of flood drainage rights based on a PSR model and entropy-based matter-element theory in the Sunan Canal, China. PLoS ONE.

[37] Sun F., Du X., Shen J. (2020). Asymmetric information barging model based on fairness-preferred trade pricing of water discharging rights. Resoueces Ind..

[38] Shen J., Li L., Zhang K., Sun F., Zhang D. (2019). Initial allocation of water discharge right based on chaos optimization-projection pursuit. Resoueces Ind..

[39] Luu C., von Meding J., Mojtahedi M. (2019). Analyzing Vietnam’s national disaster loss database for flood risk assessment using multiple linear regression-TOPSIS. Int. J. Disaster Risk Reduct..

[40] Goodarzi L., Banihabib M.E., Roozbahani A. (2019). A decision-making model for flood warning system based on ensemble forecasts. J. Hydrol..

[41] Kim T.H., Kim B., Han K. (2019). Application of Fuzzy TOPSIS to Flood Hazard Mapping for Levee Failure. Water.

[42] Song J.Y., Chung E. (2016). Robustness, Uncertainty and Sensitivity Analyses of the TOPSIS Method for Quantitative Climate Change Vulnerability: A Case Study of Flood Damage. Water Resour. Manag..

[43] Bustince H., Burillo P. (1996). Vague sets are intuitionistic fuzzy sets. Fuzzy Set Syst..

[44] Li D.F. (2005). Multiattribute decision making models and methods using intuitionistic fuzzy sets. J. Comput. Syst. Sci..

[45] Yager R.R., Abbasov A.M. (2013). Pythagorean Membership Grades, Complex Numbers, and Decision Making. Int. J. Intell. Syst..

[46] Yager R.R. (2014). Pythagorean Membership Grades in Multicriteria Decision Making. IEEE Trans. Fuzzy Syst..

[47] Zhang X., Xu Z. (2014). Extension of TOPSIS to Multiple Criteria Decision Making with Pythagorean Fuzzy Sets. Int. J. Intell. Syst..

[48] Zhang X. (2016). A Novel Approach Based on Similarity Measure for Pythagorean Fuzzy Multiple Criteria Group Decision Making. Int. J. Intell. Syst..

[49] Peng X., Yang Y. (2015). Some Results for Pythagorean Fuzzy Sets. Int. J. Intell. Syst..

[50] Zhang Y., Chen Q. (2020). Characteristics of main flood event types and their temporal-spatial variations in the upper and middle reaches of the Huai River Basin. Prog. Geogr..

[51] Du H., Xia J., Zeng S., She D., Zhang Y., Yan Z. (2012). Temporal and spatial variations and statistical models of extreme runoffin Huaihe River Basin. Acta Geogr. Sin..

[52] Zhang Y., Li Z., Liu X. (2016). Evolutionof interconnected river and lake networks in the Huai River Basin over the last millennium. South-to-North Water Transf. Water Sci. Technol..

[53] Zhang X.C., Ma C., Zhan S.F., Chen W.P. (2012). Evaluation and simulation for ecological risk based on emergy analysis and Pressure-State-Response Model in a coastal city, China. Procedia Environmental Sciences.

[54] Zhou D., Lin Z., Liu L., Zimmermann D. (2013). Assessing secondary soil salinization risk based on the PSR sustainability framework. J. Environ. Manag..

[55] Jonkman S.N., Bockarjova M., Kok M., Bernardini P. (2008). Integrated hydrodynamic and economic modelling of flood damage in the Netherlands. Ecol. Econ..

[56] Ouma Y.O., Tateishi R. (2014). Urban Flood Vulnerability and Risk Mapping Using Integrated Multi-Parametric AHP and GIS: Methodological Overview and Case Study Assessment. Water.

[57] Jin J., He J., He R., Liu C., Zhang J., Wang G., Bao Z. (2017). Impacts of Climate Change to Water Resources and Extreme Hydrological Event in the Huaihe River Basin. Sci. Geogr. Sin..

[58] Cabrera J.S., Lee H.S. (2018). Impacts of Climate Change on Flood-Prone Areas in Davao Oriental, Philippines. Water.

[59] Balica S.F., Douben N., Wright N.G. (2009). Flood vulnerability indices at varying spatial scales. Water Sci. Technol..

[60] Huo Z., Fan Y., Yang J., Shang Y. (2017). Review on agricultural flood disaster in China. J. Appl. Meteorol. Sci..

[61] Yoffe S., Wolf A.T., Giordano M. (2003). Conflict and cooperation over international freshwater resources: Indicators of basins at risk. J. Am. Water Resour. Assoc..

[62] Simonovic S.P., Nirupama (2005). A spatial multi-objective decision-making under uncertainty for water resources management. J. Hydroinform..

[63] Haider H., Ghumman A.R., Al-Salamah I.S., Ghazaw Y., Abdel-Maguid R.H. (2019). Sustainability Evaluation of Rainwater Harvesting-Based Flood Risk Management Strategies: A Multilevel Decision-Making Framework for Arid Environments. Arab. J. Sci. Eng..

[64] Li D., Zeng W., Yin Q. (2017). Distance measures of pythagorean fuzzy sets and their applications in multiattribute decision making. Control Decis..

[65] Lei Y., Hua J., Yin H., Lei Y. (2009). Technique for ascertaining nonmembership functions of intuitionistic fuzzy sets based on trichotomy. Comput. Sci..

[66] Chen Y., Yu J. (2018). Evaluation of regional ecological damage based on pythagoras TOPSIS method: A case study of the upper reaches of Yellow River Basin. Soft Sci..

[67] Kandilioti G., Makropoulos C. (2012). Preliminary flood risk assessment: The case of Athens. Nat. Hazards.

[68] Yang T., Jin Y., Yan L., Pei P. (2019). Aspirations and realities of polycentric development: Insights from multi-source data into the emerging urban form of Shanghai. Environ. Plan B Urban.

[69] Xiong L., Yan L., Li L., Jiang C., Du T. (2017). Advances in analysis of impacts of changing environments on extreme urban rainfall and drainage infrastructure. Adv. Water Sci..

